# A Combined Proteomic and Metabolomic Strategy for Allergens Characterization in Natural and Fermented *Brassica napus* Bee Pollen

**DOI:** 10.3389/fnut.2022.822033

**Published:** 2022-01-28

**Authors:** Shuting Yin, Yuxiao Tao, Yusuo Jiang, Lifeng Meng, Liuwei Zhao, Xiaofeng Xue, Qiangqiang Li, Liming Wu

**Affiliations:** ^1^Institute of Apicultural Research, Chinese Academy of Agricultural Sciences, Beijing, China; ^2^College of Animal Science, Shanxi Agricultural University, Shanxi, China

**Keywords:** *Brassica napus* bee pollen, allergens, degradation and catabolism, fermentation, *Saccharomyces cerevisiae*

## Abstract

Bee pollen is consumed for its nutritional and pharmacological benefits, but it also contains hazardous allergens which have not been identified. Here, we identified two potential allergens, glutaredoxin and oleosin-B2, in *Brassica napus* bee pollen using mass spectrometry-based proteomics analyses, and used bioinformatics to predict their antigenic epitopes. Comparison of fermented (by *Saccharomyces cerevisiae*) and unfermented bee pollen samples indicated that glutaredoxin and oleosin-B2 contents were significantly decreased following fermentation, while the contents of their major constituent oligopeptides and amino acids were significantly increased based on metabolomics analyses. Immunoblot analysis indicated that the IgE-binding affinity with extracted bee pollen proteins was also significantly decreased after fermentation, suggesting a reduction in the allergenicity of fermented bee pollen. Furthermore, fermentation apparently promoted the biosynthesis of L-valine, L-isoleucine, L-tryptophan, and L-phenylalanine, as well as their precursors or intermediates. Thus, fermentation could potentially alleviate allergenicity, while also positively affecting nutritional properties of *B. napus* bee pollen. Our findings might provide a scientific foundation for improving the safety of bee pollen products to facilitate its wider application.

**Graphical Abstract:**
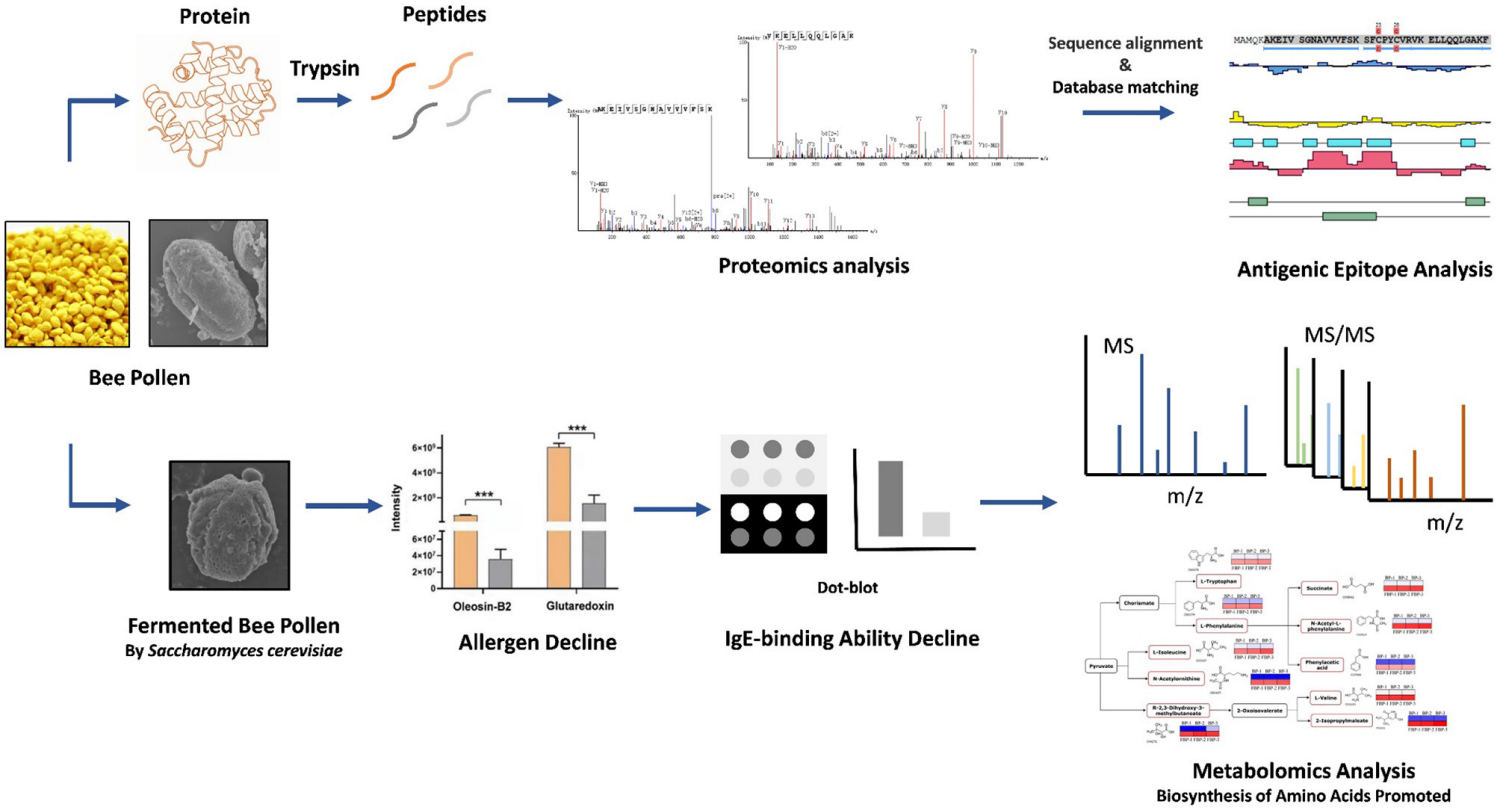


## Introduction

Bee pollen, plant pollen grains collected by honeybees and aggregated by salivary gland secretions and flower nectar, has been widely considered a complete food of abundant nutrients and bioactive compounds (1). More than 200 nutrients and bioactive components, including proteins, amino acids, carbohydrates, lipids, vitamins, minerals, polyphenols, etc. have been identified in bee pollen and its consumption reportedly confers numerous benefits that are the subject of ongoing investigation (2, 3). With increasing applications of bee pollen, for example in foods, cosmetics, and nutraceuticals, potential issues related to allergens require close scrutiny to ensure public safety. In general, patients with plant pollen allergies are also susceptible to bee pollen allergies, although some allergenic compounds in bee pollen are partially degraded or reduced by honeybee processing (4, 5).

*Brassica napus* bee pollen, which is produced worldwide, has potential sensitization hazards. Puumalainen et al. identified 2S albumins, or napins, as potential allergens in *B. napus* pollen (6). Another eight potential allergens of *B. napus* pollen were detected using immunoblot method although their primary structures have not been determined (7). However, the studies investigating allergens of bee pollen from *B. napus* remain limited, thereby resulting in a threat to its consumption.

Studies have shown that fermentation can reduce food allergens through hydrolysis (8), while improving nutritional and physicochemical characteristics of foods (9, 10). Reduction of protein allergens through fermentation has been reported for milk (11, 12), peanuts (13), soybeans (14, 15), and wheat (16, 17). Also, their nutritional properties were notably improved following fermentation, such as increasing the contents of free amino acids in milk (18), as well as improving the levels of phenolic compounds, bioactive peptides, and free amino acids in cereals (19). Fermentation was also found to improve the flavor and nutritional value of bee pollen in our previous research, such as increasing the contents of oligopeptides, fatty acids, phenolic compounds, etc. (20). However, it is unclear whether and how these effects are related to allergen degradation.

Therefore, this study aimed to identify the potential allergens in *B. napus* bee pollen using mass spectrometry-based proteomics analyses. We also conducted bioinformatic prediction of allergen epitopes for T and B cells. Furthermore, we explored the effects of fermenting *B. napus* bee pollen on its putative allergenic components, using proteomics, metabolomics, and immunoblot analyses. This research provides a scientific foundation for improving the safety of bee pollen products to facilitate its wider application.

## Materials and Methods

### Reagents

HPLC-grade methanol and acetonitrile (ACN) were purchased from Fisher Scientific (Pittsburgh, PA). Ultrapure water was purified with a Millipore Milli-Q system (Millipore, Bedford, MA). Other chemicals were purchased from Sigma-Aldrich (St. Louis., MO) except modified sequencing grade trypsin, which was obtained from Promega (Madison, WI).

### Sample Preparation

*B. napus* bee pollen samples were collected from the apiary of the Institute of Apicultural Research, Chinese Academy of Agricultural Sciences in April 2020. Samples were freeze-dried and ground into powder, and then subject to 7 kGy irradiation sterilization (IS) and stored at −80°C. The fermented bee pollen samples were prepared according to our previously reported method with several modifications (20). Briefly, 5 g sterilized bee pollen powder was mixed with 10 ml ultrapure water, then combined with 150 mg active dry *S. cerevisiae* (Angel Yeast Co., Ltd., Yichang, Hubei Province, China). The mixture was incubated at 40°C for 48 h of fermentation. Fermented samples were again freeze-dried and stored at −80°C before analysis.

### Bee Pollen Protein Pretreatment

For protein analyses, 100 mg of freeze-dried bee pollen powder was homogenized on ice for 30 min in 1 ml lysis buffer containing 8 mol/L urea, 2 mol/L thiourea, 4% 3-[(3-cholamidopropyl) dimethylammonio]-1-propanesulfonate acid (CHAPS), 20 mmol/L tris-base, and 30 mmol/L dithiothreitol (DDT). The homogenate was ultrasonicated for 90 s, then centrifugated at 12, 000 × g for 15 min at 4°C. The supernatant was collected and filtered using a Millipore 0.22 μm nylon filter, followed by addition of 5 ml ice-cold acetone for 60 min to precipitate the protein. The pellet was centrifuged at 13, 000 × g for 10 min at 4°C. The protein precipitates were collected and dried at 25 ± 2°C, then resuspended in 200 μl of 5 mol/L urea solution. Protein concentration was determined using bicinchoninic acid (BCA) protein assay kit (Beyotime biotechnology, China). Then, 100 μl protein solution was mixed with 400 μl 40 mmol/L NH_4_HCO_3_, incubated with 50 μl of 100 mmol/L DDT at 25 ± 2°C for 1 h, and alkylated with 250 μl 50 mM iodoacetamide (IAA) at 25 ± 2°C for 1 h in the dark. The acetylated protein solution was digested with modified sequencing grade trypsin by incubating at 37°C for 12 h. One microliter formic acid was added to terminate the enzymatic reaction. A Ziptip C18 column (Millipore) was used for peptide purification and enrichment. Peptide concentration was determined by Nanodrop2000, and normalized before LC-MS/MS analysis.

### Proteomics-Based Allergen Identification

Peptide samples were analyzed using an Easy-nLC1000-LTQ-Orbitrap Elite mass spectrometer (ThermoScientific, USA) with Electrospray Ionization. Prior to analytical separation, the samples were loaded onto a trap column (100 μm × 2 cm, with 5 μm Aqua C18 beads, Thermo Fisher Scientific) in mobile phase A (0.1% formic acid in ultrapure water) at a flow rate of 5 μl/min for 2 min. Then, the peptides were separated on an analysis column (75 μm × 15 cm, with 3 μm, 100 Å, Aqua C18 beads, Thermo Fisher Scientific) in a 120 min-gradient: 0 min, 3% mobile phase B (0.1% formic acid in acetonitrile); 5 min, 8% B; 85 min, 20% B; 105 min, 30% B; 110 min, 90% B; 120, 90% B, at a flow rate of 350 nl/min. The mass spectrometer was operated in positive ionization mode. Analytical parameters were identical to those in previous study (21).

The original data were collected using Xcalibur (Version 2.2, Thermo Fisher Scientific), then imported to Peaks DB 7.5 software (Bioinformatics Solutions Inc., Waterloo, Canada) for qualitative and quantitative analysis. The *Brassica* protein databases were downloaded from NCBI and Uniprot for searching and matching protein sequences. Search parameters were set as follows: precursor ion mass tolerance, 15 ppm; fragment mass tolerance, 0.05 Da; enzyme, trypsin; maximum missed cleavages, 2; maximum variable PTM per peptide, 3; fixed modification, carbamidomethyl (C, +57.02 Da); variable modification: oxidation (M, +15.99 Da). Confidently identified proteins contained at least one unique peptide with at least two spectra. False discovery rate (FDR) was set to <1% using a fusion-decoy search strategy. Quantification of relative protein abundance was conducted with Peaks Q module by a label-free quantification (LFQ) method. Triplicates of each sample were analyzed and one sample was automatically selected as representative. The quantitative parameters included: retention time offset, 0.5 s; mass error tolerance, 15 ppm. Alignment of homologous protein sequences was performed using NCBI BLAST tools based on Allergen Online Database, with reference to FAO/WHO rules (22). Antigenic epitope analysis of the potential allergens was performed with DNAStar software.

### UPLC-QTOF-MS/MS Metabolite Detection

For metabolite detection, 100 mg freeze-dried bee pollen powder was mixed with 100 μl of ultrapure water, followed by addition of 400 μl methanol. Samples were vortexed for 5 min and ultrasonicated for 10 min. After centrifugation at 13000 × g at 4°C for 15 min, supernatants were collected and filtered using an Agilent 0.22 μm nylon filter. Ten microliter of each sample was taken preparation of quality controls.

An Agilent 1,290 Infinity II series ultra-performance liquid chromatography (UPLC) system equipped with an Eclipse Plus C18 Rapid Resolution HD column (2.1 mm × 100 mm, 1.8 μm, Agilent Technologies, USA) was applied for sample separation. Mobile phases A and B were water and acetonitrile (both containing 0.1% formic acid), respectively. The gradient was set as follows: 0 min, 5% B; 2 min, 5% B; 20 min, 100% B; 25 min, 100% B; post time 5 min, with 5% B. The flow rate was 0.3 ml/min, and the injection volume was 1 μl.

An Agilent 6,545 ESI-Q-TOF system was used for MS acquisition in negative ionization mode. The mass spectrum parameters were identical to those in previous work (23). Nitrogen was used for drying, ionization, and collision. Reference ions were used to calibrate the mass accuracy. Reference ions 112.985587 and 1033.988109 were used for real-time calibration during acquisition. Detected compounds were analyzed using Agilent Mass Profiler Professional software. Metabolites were identified with the METLIN Database (DB). Metabolites with DB scores above 80 and mass error lower than 5 ppm (0.0005%) were screened as biomarkers for statistical analysis.

### Immunoblot Analysis

Bee pollen protein was extracted using a commercial plant protein extraction kit (CWBIO Co., Ltd., Jiangsu, China). Three-hundred milligram bee pollen was added with 1.5 ml protein extraction reagent, and then centrifuged at 13, 000 g for 20 min at 4°C. The supernatant was assayed by a BCA protein assay kit (CWBIO Co., Ltd., Jiangsu, China) for protein concentration quantification, and then normalized to a concentration of 25 μg/μl. Then 2 μl was loaded onto a 5 cm × 5 cm nitrocellulose membrane. After drying the membrane, it was soaked in a 10 cm culture dish containing 5% BSA solution and incubated at 25 ± 2°C for 1 h. The membrane was then incubated at 25 ± 2°C in 5% BSA solution containing 1:1000 diluted human native IgE antibody for 1 h. The membrane was washed three times with TBS-T solution for 5 min per wash, then incubated in 5% BSA solution containing HRP-labeled anti-human IgE mouse monoclonal antibody (1:1500) at 25 ± 2°C for 1 h. After washing as before, a DAB color developing kit was used following the manufacturer's instructions to reveal brown spots on the membrane. Dried, developed membrane images were captured by HP scanner (Color LaserJet Pro MFP M277dw). Image J software was used for grayscale processing, and relative quantification of dot intensity to evaluate protein binding affinity with human-specific IgE.

### Statistics

SPSS 21.0 statistical software was used for *t*-test and analysis of variance (ANOVA), conducted at 95% probability level to identify significant differences in allergenic proteins, peptides, and amino acids among samples. Multi-experiment viewer (MEV) 4.9 software was used for heat-map analysis. MetaboAnalyst 4.0 and KEGG online websites were used for metabolic pathway analysis.

## Results and Discussion

### Proteomics-Based Identification and Epitope Prediction of *B. napus* Bee Pollen Allergens

**Figure 1:**
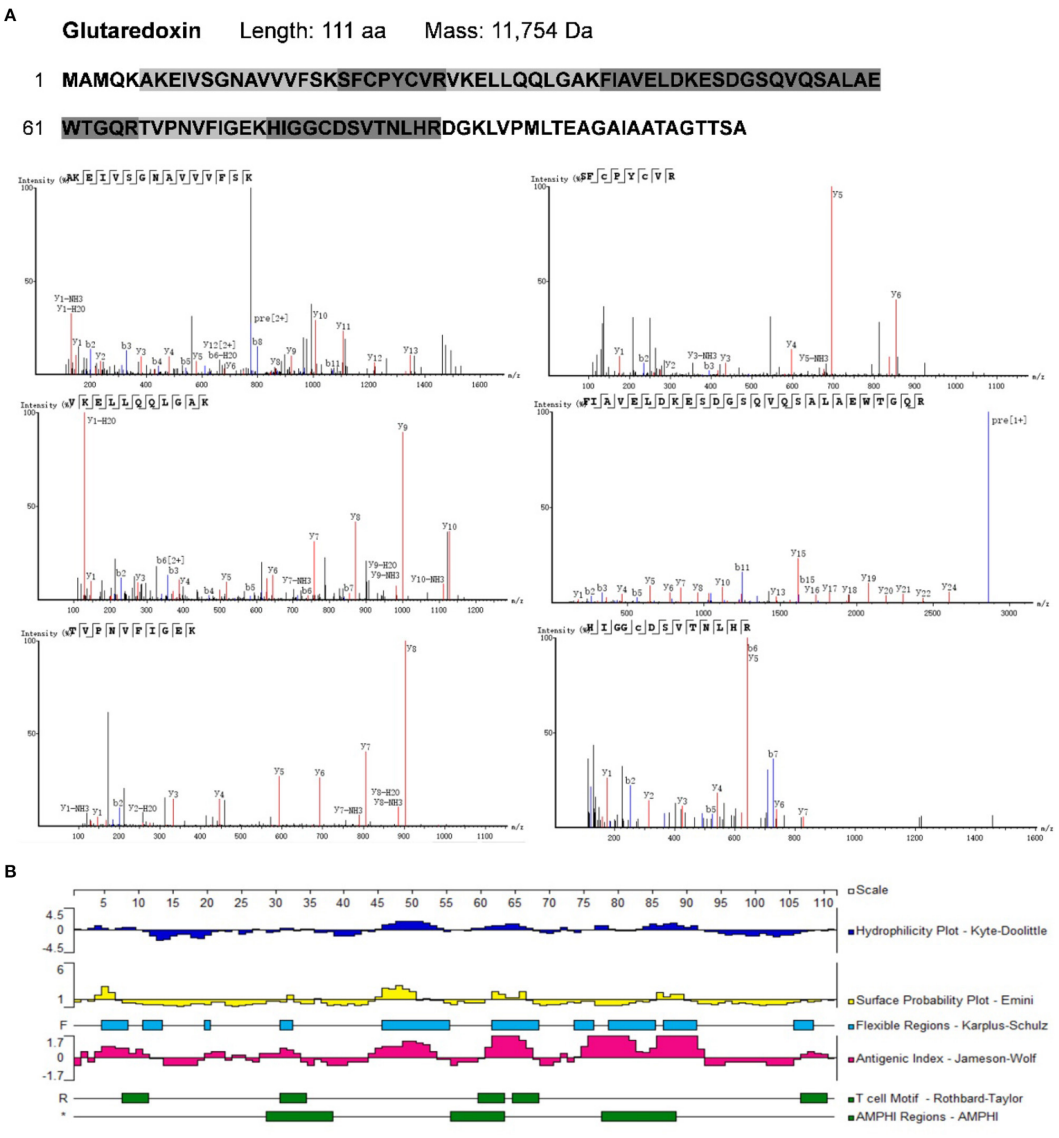
**(A)** The amino acid sequence of glutaredoxin containing the shade parts which indicated the identified amino acid sequence fragments by proteomics analysis. The mass spectra of these identified amino acid sequence fragments were displayed. **(B)** Epitope prediction of Glutaredoxin for acting with T cells and B cells.

**Figure 2:**
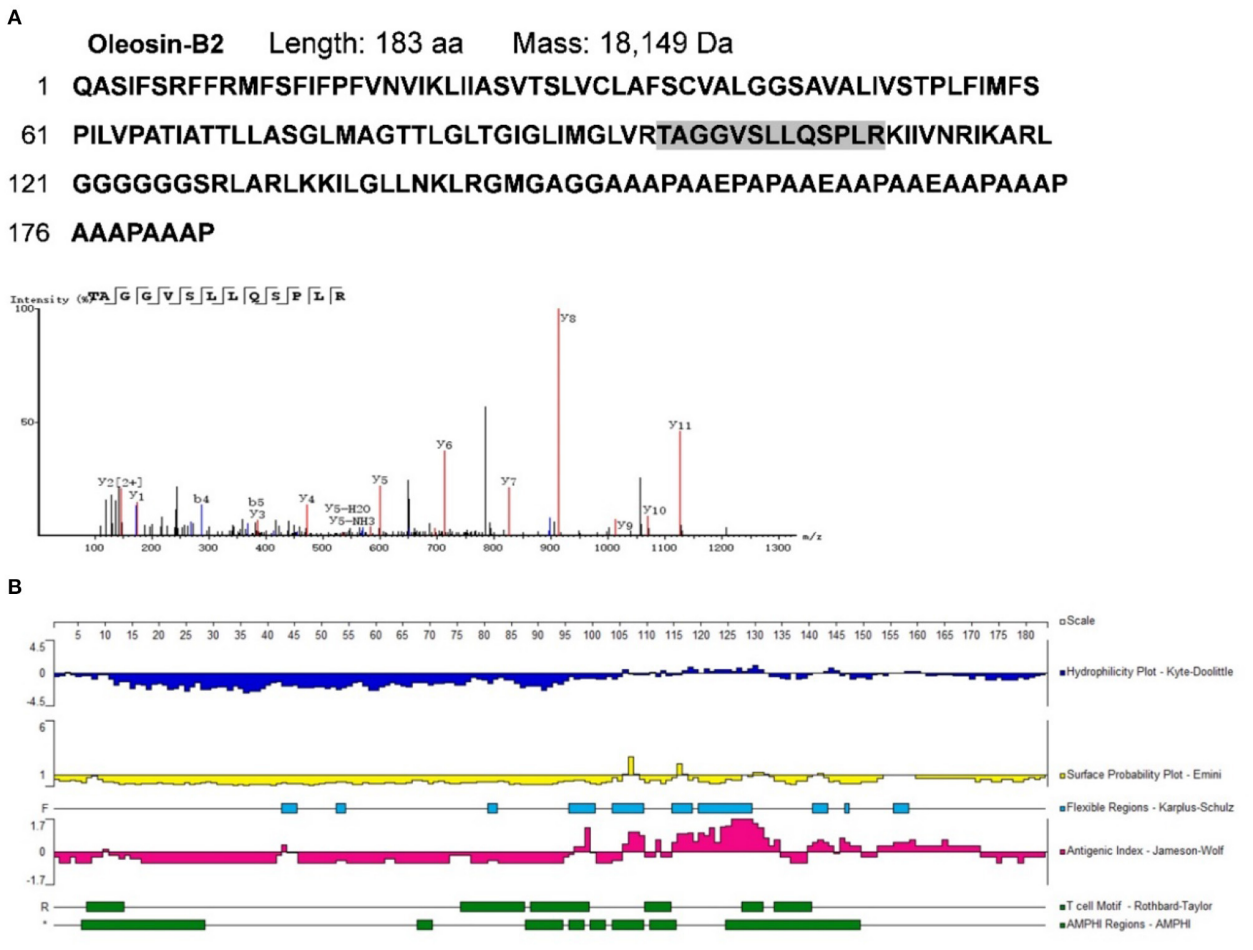
**(A)** The amino acid sequence of oleosin-B2 containing the shade part which indicated the identified amino acid sequence fragment by proteomics analysis. The mass spectrum of the identified amino acid sequence fragment was displayed. **(B)** Epitope prediction of oleosin-B2 for acting with T cells and B cells.

To identify potential allergens in *B. napus* bee pollen, we used a label-free proteomics strategy based on the NCBI and Uniprot protein databases for *Brassica*. We detected six unique peptides (AKEIVSGNAVVVFSK, VKELLQQLGAK, FIAVELDKESDGSQVQSALAEWTGQR, TVPNVFIGEK, SFCPYCVR, and HIGGCDSVTNLHR) assigned to glutaredoxin (Accession No.: A0A0D3C059) (Figure 1). Proteomics analysis showed that glutaredoxin contains 111 amino acids (MW = 11, 754 Da), and coverage of the detected peptides in the total amino acid sequence approached 75%. One unique peptide (TAGGVSLLQSPLR) assigned to oleosin-B2 (Accession No.: P29526) was also detected (Figure 2) with 183 amino acid sequence (MW = 18,149 Da) and 7% coverage of the detected peptide.

Sequence-based allergen prediction with the FAO/WHO method uses two specific comparison rules: (1) the word matching rule requires that proteins with more than six consecutive amino acids matching the sequence of a known allergen are thus considered potential allergens; (2) the sliding window matching rule requires proteins with more than 35% of any 80 consecutive amino acids that are consistent with those of a known allergen are also considered potential allergens (22, 24, 25). Redoxins represent a major class of allergens that are distributed across plants and animals, which include glutaredoxins—the thioredoxin superfamily disulfide reductases (26). As a member of thioredoxin superfamily, glutaredoxins contain a highly conserved active site C-X-X-C motifa — a major epitope region with high immunogenicity (27). Oleosins are considered typical allergens present in some allergenic plant-based foods (28). For instance, the oleosin Fag t 6 (18 kDa) from buckwheat seeds was reported to cause allergic symptoms (29). We performed the protein sequence alignment by a NCBI BLAST tool and found that oleosins-B2 has 42% homology similarity with the allergen oleosin Ara h 15 (17 kDa) from peanut. Thus, based on the FAO/WHO rules, we categorized both glutaredoxin and oleosin-B2 as potential allergens in *B. napus* bee pollen.

Previous research has led to the development of several algorithms that use the properties of amino acids (such as hydrophilicity, antigenicity, segmental mobility, flexibility, and accessibility) to predict B cell and T cell epitopes in protein sequences (30, 31). Here, we used the Protean module in DNAStar software to predict the epitopes of glutaredoxin and oleosin-B2. For glutaredoxin (Figure 1B), its potential B cell epitopes are distributed in residues 4–12, 19–23, 28–34, 44–54, 60–67, 71–82, 83–92, and 106–111, as determined by the Jameson-Wolf method. By contrast, the Rothbard-Taylor and AMPHI methods were used in conjunction to obtain the T cell epitopes of glutaredoxin located at residues 31–34 and 60–64. It showed that the predicted epitopes sites 28–34 or 31–34 were both close to the active center—C-X-X-C motifa.

For oleosin-B2 (Figure 2B), the Jameson-Wolf method identified putative B cell epitopes in residues 96–100, 106–110, 112–113, 115–135, 140–145, 146–150, and 152–172, while Rothbard-Taylor/AMPHI analyses identified T cell epitopes at amino acid positions 6–14, 88–94, 95–99, 110–115,127–132, and 134–141. Notably, antigenic regions highly overlapped with flexible regions on these peptides, indicating higher plasticity in epitope formation at these sites. Cumulatively, the findings showed that glutaredoxin and oleosin-B2 presented positive B cell and T cell epitopes, further verifying their allergenic properties.

### Degradation of Glutaredoxin and Oleosin-B2 in *B. napus* Bee Pollen Through *S. cerevisiae* Fermentation

**Figure 3:**
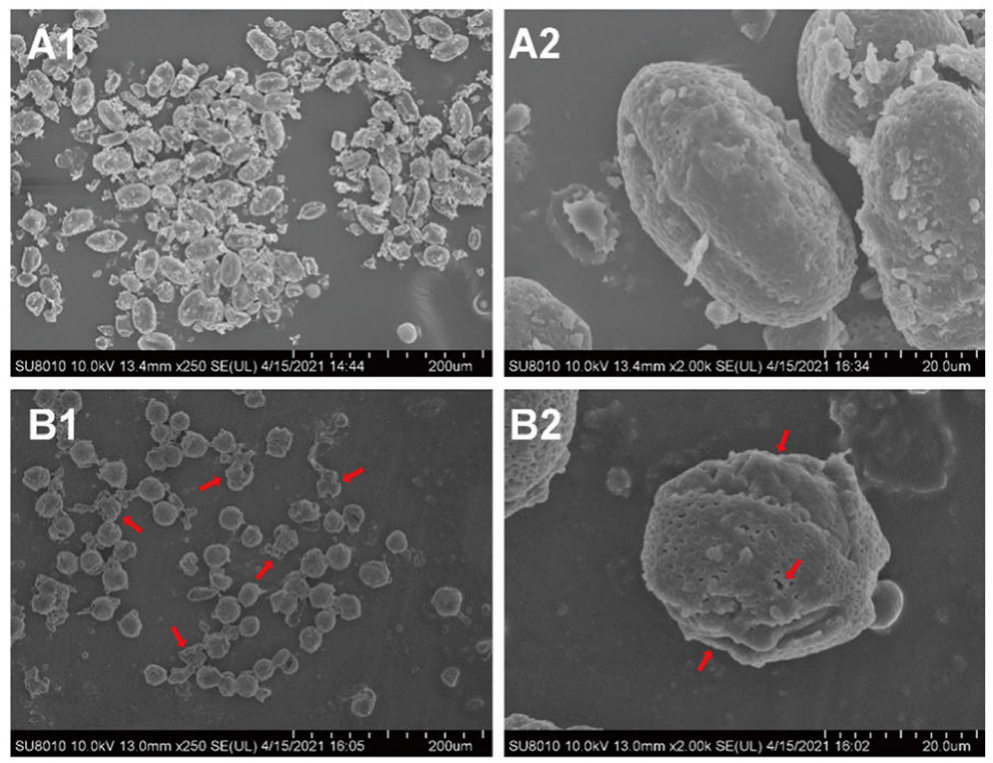
The morphological changes of *B. napus* bee pollen grains before and after fermentation by *S. cerevisiae* using scanning electron microscope (SEM). **(A1)** Unfermented *B. napus* bee pollen (250 ×). **(A2)** Single unfermented *B. napus* bee pollen grain (2000 ×). **(B1)** Fermented *B. napus* bee pollen (250 ×). **(B2)** Single fermented *B. napus* bee pollen grain (2000 ×). The red arrows indicate the breakage of outer pollen wall and the exposure of intracellular substances through fermentation.

Fermentation is a widely used method to improve the flavor and nutritional properties of bee pollen, due to lysis of pollen walls and subsequent release of pollen cell contents (20). Scanning electron microscopy (SEM) showed that *B. napus* pollen spore morphology was substantially altered by fermentation with *S. cerevisiae* (Figure 3). Before fermentation, spores were oblong, with three shallow germination grooves on the surface, and closed germination holes. Typically, the pollen outer wall exhibits a net-like, meshed pattern. After fermentation, there were obviously fewer intact spores, and pollen grains were rounder than oblong. The outer wall mesh pattern was also enlarged and sparsely distributed. In addition, pollen walls were ruptured at the germination holes with obviously leaking contents. Some spores were severely fragmented or degraded into smaller, unrecognizable components through the fermentation process.

**Figure 4:**
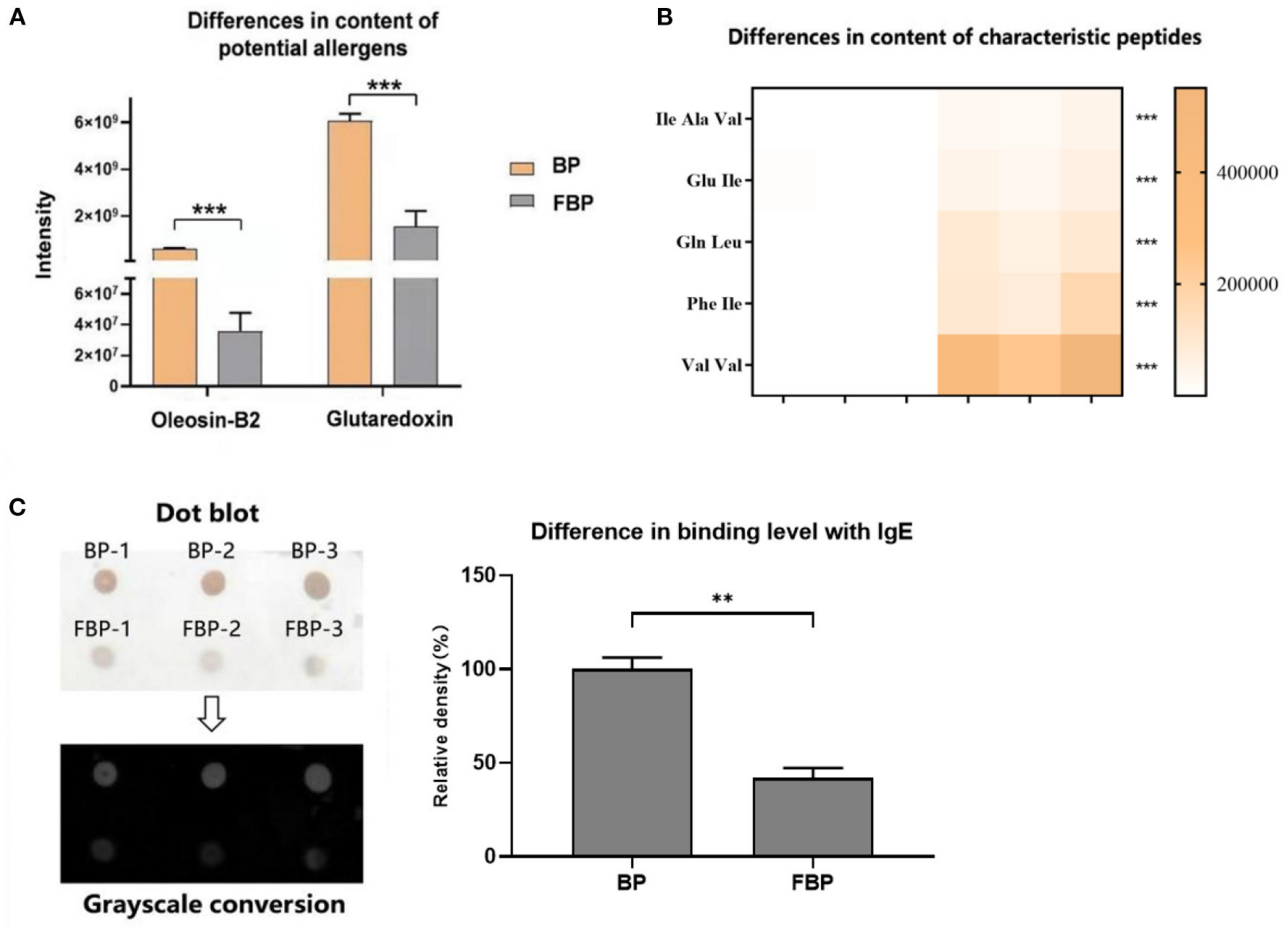
**(A)** Differences in the content of potential allergens of *B. napus* bee pollen before and after fermentation by *S. cerevisiae*. BP means the unfermented *B. napus* bee pollen, and FBP means the *S. cerevisiae* fermented *B. napus* bee pollen. **(B)** Differences in the content of characteristic oligopeptides in bee pollen before and after fermentation. The color bar from blank to yellow represented the level of five oligopeptides from low to high in the bee pollen. **(C)** Difference in the binding level of *B. napus* bee pollen protein with human native immunoglobulin E (IgE) before and after fermentation detected using Dot-blot method. Three repeated experiments were conducted. The symbol of **means *P* < 0.01, and ***means *P* < 0.001.

The major allergens in pollen are water-soluble proteins and glycoproteins located on the pollen wall (32). Enzymes secreted by yeast can destroy the pollen wall and degrade the allergens (33). Previous studies have shown that microbes can degrade allergenic proteins in food into small peptides or amino acids through fermentation, thereby reducing allergenicity (34, 35). We therefore compared the potential allergenic protein contents in *B. napus* bee pollen before and after fermentation, and found that the concentrations of glutaredoxin and oleosin-B2 were significantly lower in fermented samples compared with those of unfermented samples (*P* < 0.001) (Figure 4A). In addition, metabolomics analysis revealed that the contents of five oligopeptides, including Ile Ala Val, Glu Ile, Gln Leu, Phe Ile, and Val Val, substantially increased in fermented samples compared to unfermented samples (Figure 4B). Furthermore, the individual contents of L-valine, L-isoleucine, L-tryptophan, and L-phenylalanine were also increased in the fermented pollen samples (Table 1). Together, these five oligopeptides and four amino acids represent key constituent fractions of glutaredoxin and oleosin-B2, and the commensurate increase in their levels was closely correlated with the observed decreases in glutaredoxin and oleosin-B2, suggesting that yeast-based fermentation could degrade these putative allergens into oligopeptides and amino acids.

**Table 1:** The metabolic pathway analysis base on the changed metabolites in fermented bee pollen by *S. cerevisiae*.

**Pathway**	**Compound**	**KEGG ID**	**Formula**	**Mass**	**Score (DB)**	**FC ([Y] Vs [C])**	**Regulation**	***P*-value**
Biosynthesis of	L-Valine	C00183	C5 H11 N O2	117.0789	88	4.637	Up	^***^
amino acids	L-Isoleucine	C00407	C6 H13 N O2	131.0943	88	10.098	Up	^***^
	2,3-Dihydroxy-3-methylbutyric acid	C04272	C5 H10 O4	134.0582	99	37142.844	Up	**
	L-Tryptophan	C00078	C11 H12 N2 O2	204.0903	98	4.535	Up	^***^
	L-Phenylalanine	C00079	C9 H11 N O2	165.0794	99	68.297	Up	^***^
	N2-Acetyl-L-ornithine	C00437	C7 H14 N2 O3	174.1007	99	341288.970	Up	^***^
Valine, leucine	L-Isoleucine	C00407	C6 H13 N O2	131.0943	88	10.098	Up	^***^
and isoleucine	L-Valine	C00183	C5 H11 N O2	117.0789	88	4.637	Up	^***^
biosynthesis	2,3-Dihydroxy-3-methylbutyric acid	C04272	C5 H10 O4	134.0582	99	37142.844	Up	^**^
	2-Isopropylmaleate	C02631	C7 H10 O4	158.0576	99	33276.340	Up	^***^
Phenylalanine	Phenylacetic acid	C07086	C8 H8 O2	136.0520	85	5980.313	Up	^***^
metabolism	Succinic	C00042	C4 H6 O4	118.0271	98	9.355	Up	^***^
	N-Acetyl-L-phenylalanine	C03519	C11 H13 N O3	207.0894	99	16.499	Up	^***^
	L-Phenylalanine	C00079	C9 H11 N O2	165.0794	99	68.297	Up	^***^

** *means P < 0.01;*

*** *means P < 0.001*.

### Fermentation Reduced Binding Affinity Between Fermented Pollen Proteins and IgE

We next used dot-blot assays to observe and quantify binding affinity between proteins extracted from fermented or unfermented *B. napus* bee pollen and human IgE. The results showed a coefficient of variation of <10% among replicate dots, suggesting low variability. After grayscale conversion and analysis of the integrated intensity of each dot, we found that fermented pollen proteins exhibited significantly lower binding affinity with IgE compared to that of unfermented pollen proteins (*P* < 0.001) (Figure 4C).

This finding was in agreement with reports showing that the majority of food allergies cause type I hypersensitivity, mediated by IgE, which induces degranulation of sensitized mast cells or basophils (36). When specific allergens first enter the human digestive tract, they are processed by dendritic cells, then delivered to Th2 cells. Th2 cells subsequently induce B proliferation by secreting IL-4, IL-13, and other cytokines, and produce IgE antibody. The Fc fragment of IgE binds to the Fc high-affinity receptor (FcεRI) on the surface of mast cells or basophils resulting in sensitization. Upon subsequent exposure to the same allergen, it specifically binds to IgE, inducing mast cells or basophils to release vasoactive amines and cytokines, heightening the allergic reaction (37). Food allergens include both linear and conformational epitopes to IgE. Conformational epitopes are easily denatured during food processing, whereas changes in linear epitopes require manipulation of specific residues in the peptide (38). Our results suggest that fermentation by yeast can potentially degrade allergens into peptides and single amino acids, thereby destroying linear epitopes of IgE, and ultimately resulting in decreased binding between allergens and IgE. This effect was consistent with the decrease in potentially allergenic proteins and increase in constituent fragments detected in the fermented bee pollen. Collectively, these results suggest that allergenicity of bee pollen could be reduced through fermentation by *S. cerevisiae*.

### Intervention of *S. cerevisiae* in Amino Acid Metabolism

**Figure 5:**
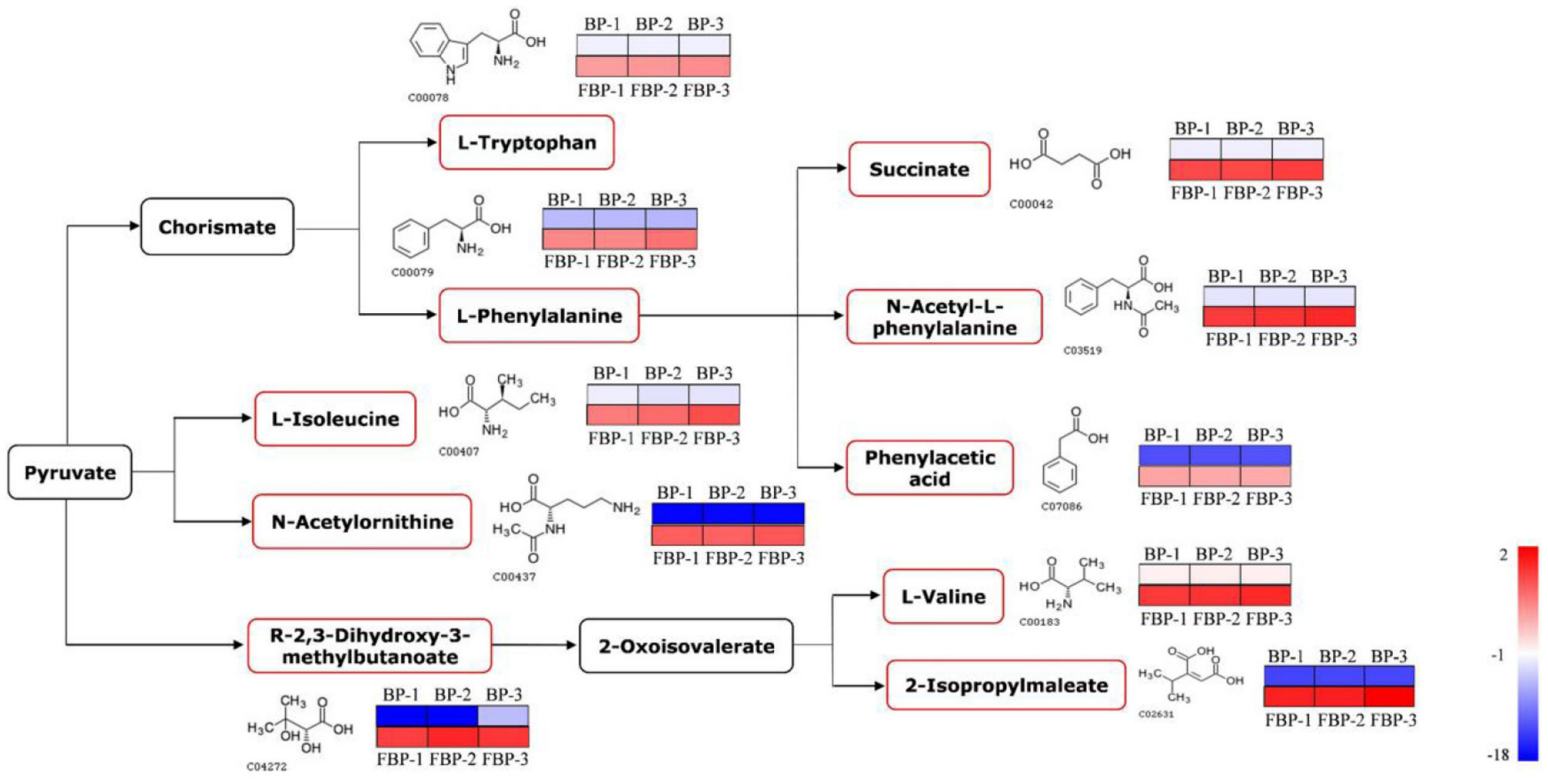
The changed metabolic pathways due to *S. cerevisiae* fermentation in *B. napus* bee pollen based on KEGG pathway analysis. The red frame represented that the content of those highlighted metabolites was significantly increased in the fermented bee pollen compared to the unfermented bee pollen. The color bar from blue to red indicated the metabolite level from low to high.

In addition to proteins, we detected the metabolites that accumulated to significantly different levels (*P* < 0.05; Fold Change>2) between fermented and unfermented bee pollen (Table 1). KEGG pathway enrichment analysis suggested that the most significantly enriched metabolites were involved in three main pathways: (1) biosynthesis of amino acids, (2) valine, leucine and isoleucine biosynthesis, and (3) phenylalanine metabolism (Figure 5). These metabolites were all found in higher concentrations in the fermented bee pollen, which suggested that fermentation promoted the biosynthesis of L-valine, L-isoleucine, L-tryptophan, and L-phenylalanine.

The branched-chain amino acids L-valine and L-isoleucine are essential to humans, which can maintain blood glucose level, supply energy, and protect muscle function (39–41). The aromatic amino acids L-tryptophan and L-phenylalanine, which cannot be synthesized by humans but are essential for protein synthesis, are known to regulate the levels of insulin-like growth factor-1 and leptin (42). Additionally, 2,3-Dihydroxy-3-methylbutyric acid can be converted into 2-oxoisovalerate through dehydration, which is a substrate for L-valine and 2-isopropylmaleate production by transaminase. 2-Isopropylmaleate is involved in the synthesis of L-leucine, another branched-chain amino acid that promotes myofibrillar protein synthesis and metabolism (43). Fermentation apparently increased the contents of these essential amino acids and their precursors or intermediates in bee pollen, suggesting that this process improved nutritional properties while also reducing allergenicity of *B. napus* bee pollen.

## Conclusion

Through proteomics analysis, we identified two putative allergenic proteins, glutaredoxin and oleosin-B2 in *B. napus* bee pollen, and predicted their antigenic epitopes to B and T cells. We also found differences in the levels of allergenic proteins, characteristic peptides, and predominant amino acids between unfermented and fermented bee pollen. Notably, glutaredoxin and oleosin-B2 contents were significantly lower, while their five major constituent oligopeptides and four amino acids were significantly higher in fermented bee pollen compared to that in unfermented pollen samples, which indicated fermentation by *S. cerevisiae* could degrade these two potential allergens. Immunoblot analysis showed that the IgE-binding affinity of fermented bee pollen protein extract also declined, suggesting an apparent decrease in allergenicity attributable to the fermentation process. Metabolomics analysis revealed that fermentation apparently promoted the biosynthesis of essential amino acids, implying that *S. cerevisiae* fermentation also exerted positive effects on the nutritional properties of *B. napus* bee pollen. The findings provide a scientific foundation for improving the safety of bee pollen products to facilitate its wider application.

## Data Availability Statement

The datasets presented in this study can be found in online repositories. The names of the repository/repositories and accession number(s) can be found at: http://proteomecentral.proteomexchange.org PXD030273; https://www.ebi.ac.uk/metabolights/ MTBLS3950.

## Author Contributions

SL and YT: writing-original draft preparation, investigation, and formal analysis. YJ, LM, LZ, and XX: validation. QL: writing-original draft preparation, conceptualization, methodology, writing-reviewing and editing, supervision, project administration, and funding acquisition. LW: supervision, project administration, and funding acquisition. All authors contributed to the article and approved the submitted version.

## Funding

This work was supported by the National Natural Science Foundation of China (No. 32102605), and the Agricultural Science and Technology Innovation Program under Grant (CAAS-ASTIP-2020-IAR).

## Conflict of Interest

The authors declare that the research was conducted in the absence of any commercial or financial relationships that could be construed as a potential conflict of interest.

## Publisher's Note

All claims expressed in this article are solely those of the authors and do not necessarily represent those of their affiliated organizations, or those of the publisher, the editors and the reviewers. Any product that may be evaluated in this article, or claim that may be made by its manufacturer, is not guaranteed or endorsed by the publisher.
